# Authors’ reply

**Published:** 2011-02

**Authors:** Harminder Singh, Navin Dulhani, Bithika Nel Kumar, Prabhakar Singh, Pawan Tiwari

**Affiliations:** Department of Pharmacology, Government Medical College, Jagdalpur, Chhattisgarh, India

Sir,

This is with reference to the comments on our article.[1,2] Respected reader has raised his/her concern on the criteria that merited its publication. In support of our published article, we humbly put forward the following:

1. The established drug efficacy studies/concepts need repetition in different gene pool/geographical area to verify the optimal results in diverse situations.[3,4] The repeated validation in dissimilar condition makes the established fact more relevant.
2. Apart from the efficacy of hydroxyurea, we also had our concern about safety aspects throughout the study period.[2]
3. Even the negative results are welcome in scientific community and some of the journals are fanatical for this job. We have conducted a scientifically sound, ethically approved, prospective study in sickle cell disease endemic tribal area which itself holds some logical merit to make a place in indexed journal.[5]
4. Our main objective was to recommend a wider adoption of hydroxyurea for treatment in areas of high prevalence of sickle cell disease, which is still lacking. The *Indian Journal of Pharmacology* has shown a full accountability and scientific conclusion to decide on this study in their much valued journal.
